# Population Genetic Structure and Regional Divergence of the Endangered Freshwater Fish Black Shinner *Pseudopungtungia nigra* Based on Mitochondrial DNA

**DOI:** 10.3390/biology15110833

**Published:** 2026-05-26

**Authors:** Kang-Rae Kim, In-Chul Bang

**Affiliations:** 1 Southeast Sea Fisheries Research Institute, National Institute of Fisheries Science, Tongyeong-si 52440, Republic of Korea; kimkangrae9586@gmail.com; 2 Department of Life Science, Soonchunhyang University, Asan 31538, Republic of Korea

**Keywords:** *Pseudopungtungia nigra*, mitochondrial DNA, cytochrome b, genetic diversity, population structure, haplotype network

## Abstract

Assessing genetic structure is important for conserving endangered freshwater fishes inhabiting fragmented river systems. *Pseudopungtungia nigra*, a Korean endemic species, occurs in several isolated drainages, but its mitochondrial population structure has not been fully evaluated. In this study, we analyzed mitochondrial cytochrome b (*cytb*) sequences from 80 individuals across eight populations to examine genetic diversity, haplotype composition, and population differentiation. A total of 25 haplotypes were detected, indicating relatively high diversity at the species level. However, diversity was uneven among populations: the Mangyeonggang (MG) population contained only two haplotypes, both of which were not found in the other populations, and showed the lowest haplotype and nucleotide diversity among the sampled populations. Multiple analyses, including pairwise differentiation, haplotype network reconstruction, principal coordinates analysis, and AMOVA, consistently identified MG as the most divergent population. The mitochondrial pattern was also concordant with previously reported microsatellite-based structure, supporting a major division between MG and the remaining populations. These findings indicate that *P. nigra* preserves substantial diversity overall, whereas the MG population showed a restricted and population-specific *cytb* haplotype composition. This study provides a genetic basis for defining conservation units and for guiding restoration and management strategies in this endangered species.

## Simple Summary

Understanding genetic structure is essential for conserving endangered freshwater fishes that occur in fragmented river systems. In this study, we analyzed mitochondrial cytochrome b sequences from eight populations of the Korean endemically endangered fish *Pseudopungtungia nigra* to assess genetic diversity and population differentiation. Overall, the species retained relatively high haplotype diversity, but the Mangyeonggang (MG) population showed markedly reduced diversity and strong genetic distinctiveness from the other populations. Multiple analytical approaches, including pairwise differentiation, haplotype network analysis, PCoA, and AMOVA, consistently supported this pattern. These mitochondrial results were also concordant with previously reported microsatellite-based population structure, indicating that the divergence of the MG population is not marker-specific but reflects historically differentiated lineages. Our findings provide important baseline information for defining conservation units and for avoiding inappropriate mixing of genetically distinct populations in future restoration and management programs.

## 1. Introduction

Freshwater fishes are among the most vulnerable vertebrates because their populations are often restricted to isolated drainages and are strongly affected by habitat fragmentation, dam construction, pollution, and local extinction [1,2]. River fragmentation can reduce dispersal among tributaries and drainage basins, thereby limiting gene flow among local populations [3]. Over time, such restricted connectivity can increase the effects of genetic drift and promote differentiation in haplotype composition and allele frequencies [4]. In endangered freshwater fishes, genetic markers are therefore essential for identifying population subdivision, quantifying genetic diversity, and defining conservation units [5,6]. Microsatellite markers are particularly useful for detecting recent gene flow, heterozygosity, inbreeding, and fine-scale population structure, whereas mitochondrial markers are informative for tracing lineages, historical isolation, and phylogeographic divergence [7,8]. Because these marker systems reflect different inheritance modes and temporal scales, their combined use provides a more complete understanding of both contemporary and historical population processes [9,10]. Similar conservation-oriented applications of mitochondrial and microsatellite markers have also been reported in other fragmented or threatened freshwater taxa, highlighting their value for detecting population isolation, historical divergence, and management-relevant structure [11,12,13].

The black shinner *Pseudopungtungia nigra* Mori, 1935 (Cypriniformes: Gobionidae), is an endangered freshwater fish endemic to Korea, with a narrow distribution limited to the Geumgang River system, Mangyeonggang River, and Ungcheoncheon Stream [14]. Because the species inhabits fragmented river systems and has experienced habitat loss and restoration, understanding its genetic structure is essential for conservation planning [5,14]. A previous microsatellite study of *P. nigra* using 21 loci demonstrated moderate genetic diversity overall, but lower diversity in the Mangyeonggang population, significant bottleneck signals, and clear separation of the Mangyeonggang population from the Geumgang and Ungcheoncheon (UC) populations [14]. However, because that study was based on nuclear microsatellite markers, it did not evaluate whether the same population structure is also reflected in mitochondrial haplotype composition [14]. That study further showed that the major nuclear genetic structure was best explained by two clusters and that the UC population was genetically closer to the Geumgang system than to the Mangyeonggang population [14]. Clarifying whether these geographically separated populations represent shallow contemporary subdivisions or historically differentiated lineages is important for defining conservation units and for avoiding inappropriate mixing among populations during future management or restoration efforts [5,15]. To date, however, a population-wide assessment of *cytb* haplotype diversity and mitochondrial structure in *P. nigra* has remained lacking [14].

Accordingly, mitochondrial DNA analysis is needed to determine whether the nuclear structure detected in the previous microsatellite study is also reflected in *cytb* haplotype composition and sequence divergence, which can be strongly influenced by drainage isolation in freshwater fishes [8,16,17,18,19,20,21]. Concordance or discordance between microsatellite and mtDNA patterns can therefore help determine whether the Geumgang-Mangyeonggang division is consistently supported across marker systems or reflects processes such as recent admixture, incomplete lineage sorting, or marker-specific temporal sensitivity [9,10,15,17,18,19,20,22]. Thus, a mitochondrial approach offers an opportunity to add historical and phylogeographic depth to the population structure already identified from microsatellites [9,16,17,18,21]. This issue is particularly relevant in *P. nigra*, whose narrow distribution across isolated western Korean drainages makes the species especially susceptible to lineage divergence under long-term hydrological separation [14].

Among mitochondrial markers, cytochrome b (*cytb*) has been widely used in population genetic and phylogeographic studies of freshwater fishes because it provides an appropriate balance between sequence conservation and intraspecific variation [23,24,25]. This marker is particularly useful for resolving haplotype relationships and detecting historical population subdivision at the lineage level [26,27,28]. In the present study, *cytb* was selected because it was expected to provide sufficient haplotype resolution for evaluating mitochondrial diversity and population structure in *P. nigra*, while offering a more suitable balance between population-level variability and alignment reliability than more conserved COI or highly variable control-region markers [17,28].

In the present study, we analyzed mitochondrial *cytb* sequences from eight populations of *P. nigra* to evaluate genetic diversity, haplotype composition, and population structure. Based on the previous microsatellite study and the geographic isolation of the Mangyeonggang River, we expected that the Mangyeonggang population might show relatively reduced *cytb* haplotype diversity, a more population-specific haplotype composition, and stronger mitochondrial differentiation from the remaining populations. We used these expectations to assess whether the population structure previously detected with microsatellite markers is also reflected in mitochondrial *cytb* variation. By integrating the present mtDNA results with previous nuclear marker-based findings, this study provides baseline genetic information for understanding population structure and guiding conservation-unit designation in *P. nigra*.

## 2. Materials and Methods

### 2.1. Sampling and Genomic DNA Extraction

**Figure 1:**
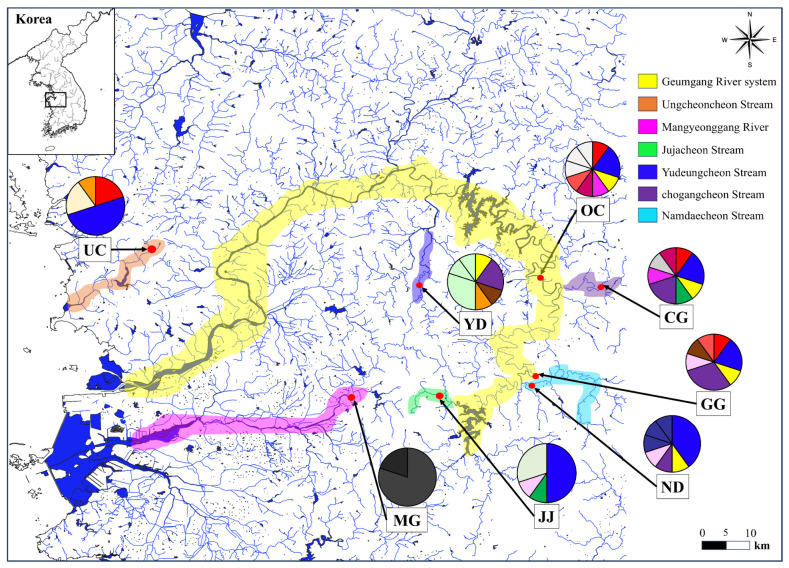
Sampling locations and hydrographic distribution of *P. nigra* populations in Korea. Map showing the sampling locations of eight *P. nigra* populations and the associated river systems in Korea. Red dots indicate the sampling sites, labeled as UC, MG, JJ, YD, OC, CG, GG, and ND. The red circular shape connected to the black arrow is the collection site. Colored shaded areas represent the major river systems or streams associated with the distribution of the sampled populations: the Geumgang River system (yellow), Ungcheoncheon Stream (orange), Mangyeonggang River (magenta), Jujacheon Stream (green), Yudeungcheon Stream (blue), Chogangcheon Stream (purple), and Namdaecheon Stream (cyan). Blue lines indicate river channels and tributaries. The inset map in the upper left shows the location of the study area within Korea. Scale bar and compass rose are provided in the lower right and upper right corners, respectively. Pie charts indicate the relative frequencies of *cytb* haplotypes in each population. Each color in the pie charts represents a different haplotype, and identical colors across pie charts represent the same haplotype. To avoid overcrowding the figure, haplotype IDs were not labeled directly within the pie-chart segments; the haplotype IDs corresponding to each population are provided in the accompanying haplotype information and described in the Section 3. Identical colors across pie charts represent the same haplotype. The river network and base map were prepared in QGIS (https://qgis.org, accessed on 1 January 2026) using publicly available spatial datasets, and the highlighted river systems indicate the major drainage areas associated with the sampled populations.

A total of 80 preserved tissue samples of *Pseudopungtungia nigra*, representing eight populations with 10 individuals per population, were used in this study, and detailed sampling information is provided in Figure 1 and Table S1. These samples had been collected between April 2018 and 2019 under national collection permits and subsequently preserved at Soonchunhyang University. Because *P. nigra* is an endangered species, the number of individuals sampled from each population was limited to minimize disturbance to natural populations. We therefore used an equal sample size of 10 individuals per population, which allowed standardized comparison of haplotype diversity and population differentiation across the eight populations. The original field collection and handling of this legally protected species were authorized by the Ministry of Environment, Korea (permit nos.: 2018-35, 2019-26, 2018-16, and 2019-15), and the use of preserved samples for the present molecular analysis was conducted in accordance with relevant institutional and national guidelines. The preserved fin tissues were stored in ethanol at low temperature until genomic DNA extraction for the present study. Genomic DNA was extracted using the HiGene^TM^ Genomic DNA Prep Kit for Animal Tissue (BioFact, Daejeon, Republic of Korea) following the manufacturer’s protocol.

### 2.2. mtDNA Sequencing and Sequence Assembly

For analysis of mitochondrial variation, a fragment of the cytochrome b (*cytb*) gene was selected because it provided an appropriate level of haplotype diversity. Primers for the mitochondrial *cytb* gene were designed from conserved flanking regions suitable for *cytb* amplification. Candidate primers were evaluated using PrimerStats (https://www.bioinformatics.org/sms2/pcr_primer_stats.html; accessed on 10 April 2020) to assess primer properties, including melting temperature, GC content, and PCR suitability. Amplification of the *cytb* region was carried out with the primer pair *P. nigra*_cytb_F2 (5′-GAACAATAATGGCAAGCCTACGA-3′) and *P. nigra*_cytb_R2 (5′-CTAAGCTACTAGGGCAAGCTC-3′) using a Mastercycler^®^ pro thermal cycler (Eppendorf AG, Hamburg, Germany). The primer pair amplified a fragment of about 1220 bp containing the entire mitochondrial cytb region and part of the tRNA region. Each PCR reaction was performed with AccuPower^®^ PCR Premix (BIONEER Co., Daejeon, Republic of Korea) and consisted of 50 ng/μL of genomic DNA template, 1 μL of each primer (1.0 μM), and 17 μL of tertiary distilled water. Thermal cycling was initiated with an initial denaturation step at 95 °C for 5 min, followed by 34 cycles of 94 °C for 30 s, 57 °C for 30 s, and 72 °C for 30 s, with a final extension at 72 °C for 10 min and a holding step at 4 °C. Amplified products were sequenced on an ABI 3730xl DNA Analyzer (Applied Biosystems, Waltham, MA, USA). Forward and reverse chromatograms were visually inspected in Geneious Prime 2026.0.2 [29]. Low-quality terminal regions were trimmed, and ambiguous base calls were checked manually. Consensus sequences were generated only when reliable sequence signals were obtained from both directions, and only unambiguous aligned sites were retained for downstream analyses. The resulting *cytb* haplotype sequences are provided in FASTA format in the Supplementary Data and were deposited in GenBank under accession numbers PZ315716-PZ315740.

### 2.3. Sequence Analysis of Genetic Diversity and Structure in cytb of mtDNA

The *cytb* sequences were aligned using the MAFFT algorithm in Geneious Prime 2022.2 [29,30]. Genetic diversity was characterized by estimating the number of haplotypes, haplotype diversity (*H*_d_), nucleotide diversity (π), Fu’s Fs (*F*), and Tajima’s D (*D*) in DnaSP version 5.0 [31]. Haplotype relationships were inferred using a median-joining network constructed in Network version 10.2.0.0 [32]. To further visualize genetic relationships among individuals and populations, principal coordinates analysis (PCoA) was performed in R (version 4.5.2) based on a pairwise raw *P*-distance matrix calculated from aligned *cytb* sequences using the ape package (version 5.8-1) [33]. The first two coordinate axes were obtained by classical multidimensional scaling (cmdscale), and the percentage of explained variation for each axis was calculated from the positive eigenvalues. The ordination results were visualized in ggplot2 (version 4.0.0) [34] using population-specific colors and symbols.

To evaluate population structure, overall genetic differentiation among populations was examined in DnaSP version 5.0 [31] using the Chi-square, Hst, Kst, Kst*, Z, Z*, and Snn statistics, with significance assessed by 50,000 permutation replicates. Pairwise differentiation among populations was further estimated as *F*_ST_ in ARLEQUIN version 3.5 [35]. Population divergence was also assessed using Dxy, the average number of nucleotide substitutions per site between populations, and Da, the net nucleotide divergence corrected for within-population diversity [31].

Mismatch distribution analysis was performed for each population using *cytb* sequence data in DnaSP version 5.0 [31] The observed distribution of pairwise nucleotide differences was compared with the expected unimodal distribution under a sudden expansion model. This analysis was used to evaluate whether the demographic history of each population was more consistent with recent expansion or with long-term demographic stability.

AMOVA was conducted in ARLEQUIN version 3.5 [35] to partition molecular variation among groups, among populations within groups, and within populations. In the AMOVA analysis, MG was classified into one group, and the remaining populations into a second group. This grouping was based on the *F*_ST_ and haplotype network differentiation patterns observed in the *cytb* dataset. The significance of fixation indices and variance components was evaluated using 50,000 permutations.

## 3. Results

### 3.1. Genetic Diversity of Mitochondrial cytb

A total of 80 individuals from eight populations of *P. nigra* yielded 25 *cytb* haplotypes (Table 1). Overall haplotype diversity was high (*H*_d_ = 0.910 ± 0.021), whereas nucleotide diversity was moderate to low (π = 0.00409 ± 0.00024). Among populations, haplotype diversity ranged from 0.356 ± 0.159 in MG to 0.978 ± 0.054 in OC, and nucleotide diversity ranged from 0.00062 ± 0.00028 in MG to 0.00432 ± 0.00072 in YD. OC showed the highest number of haplotypes (*h* = 9), followed by CG (*h* = 8), whereas MG showed the lowest haplotype richness (*h* = 2), together with the lowest haplotype and nucleotide diversity.

Neutrality statistics varied among populations, but none showed a strongly consistent signal of recent demographic expansion across all indices. Tajima’s D was negative in CG, GG, ND, OC, and YD, but positive in JJ, MG, and UC. Fu’s Fs was most strongly negative in OC (−4.031) and CG (−2.785), whereas positive values were observed in JJ, MG, and UC. Fu and Li’s D and F showed a similar pattern, with negative values in CG, GG, ND, OC, and YD and positive values in JJ, MG, and UC. Overall, these results indicate that mitochondrial diversity was unevenly distributed among populations, with MG showing markedly reduced variation relative to the other populations. Because only 10 individuals were analyzed per population and several populations had few segregating sites, the neutrality tests may have limited statistical power.

### 3.2. Genetic Structure of Population

Overall genetic differentiation among the eight populations was highly significant for the *cytb* dataset (Table 2). All overall differentiation statistics were significant at *p* < 0.001, indicating significant mitochondrial population structure among the eight populations. The significant Chi-square and Hst values suggest differences in haplotype composition among populations, whereas the significant Kst, Kst, Z, Z, and Snn values indicate that this differentiation was also supported by sequence-based comparisons.

**Figure 2:**
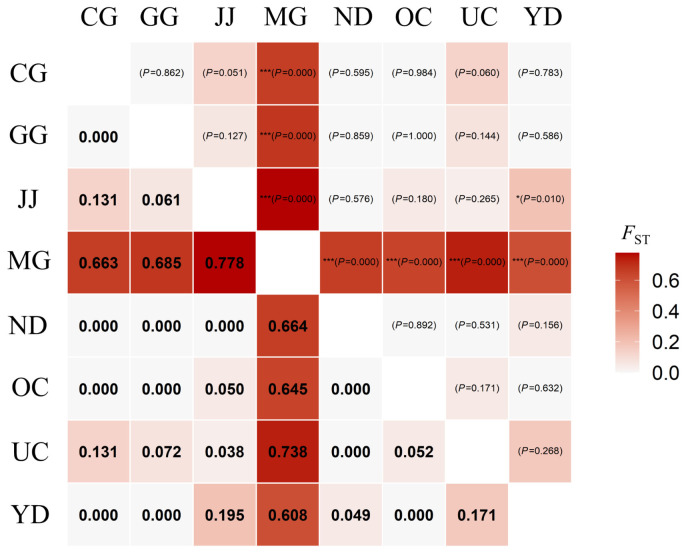
Pairwise *F*_ST_ values and significance of *cytb* differentiation among eight populations of *P. nigra*. Heatmap showing pairwise genetic differentiation among eight populations of *P. nigra* based on mitochondrial *cytb* sequences. Lower triangular cells present pairwise *F*_ST_ values, whereas upper triangular cells show the corresponding significance levels as permutation-based *p*-values. Color intensity reflects the magnitude of pairwise *F*_ST_, with darker red indicating stronger genetic differentiation. Population codes are shown along both axes. Asterisks indicate levels of statistical significance: * *p* < 0.05; *** *p* < 0.001.

The MG population was strongly differentiated from all other populations, with pairwise *F*_ST_ values ranging from 0.608 to 0.778, and all comparisons involving MG were highly significant (*p* = 0.000; Figure 2). In contrast, several comparisons among the remaining populations showed little or no differentiation, with *F*_ST_ values of 0.000 between CG and GG, CG and ND, GG and ND, GG and OC, and GG and YD. Moderate differentiation was observed in some population pairs, including JJ-YD (*F*_ST_ = 0.195), UC-YD (*F*_ST_ = 0.171), and CG-JJ and CG-UC (both *F*_ST_ = 0.131).

**Figure 3:**
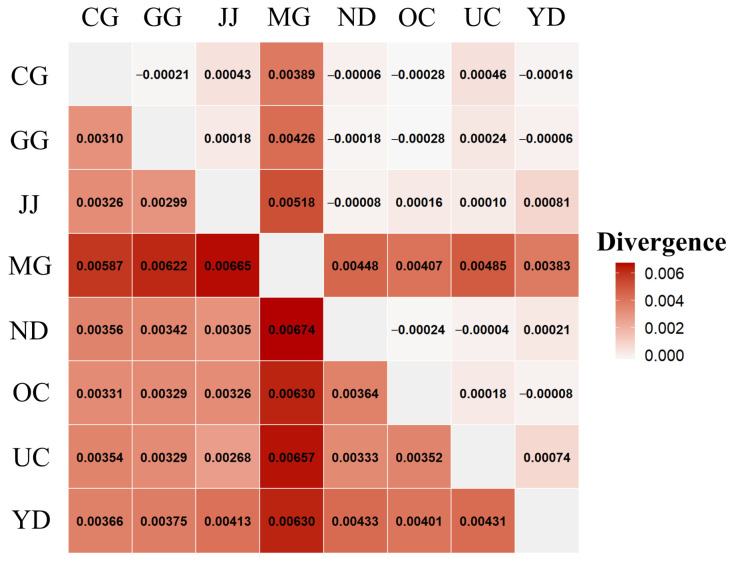
Heatmap of pairwise Dxy and Da values among populations of *P. nigra* based on mitochondrial *cytb*. Lower triangular cells indicate Dxy values, whereas upper triangular cells indicate Da values. Dxy represents the average number of nucleotide substitutions per site between populations, and Da represents the net nucleotide divergence after correction for within-population diversity. The color scale reflects the magnitude of divergence, with darker red indicating greater sequence differentiation. Slightly negative Da values may occur when between-population divergence is extremely low relative to within-population variation.

Dxy and Da values were generally highest in comparisons involving MG (Figure 3). The highest Dxy was observed between ND and MG (0.00674), and the highest Da was observed between JJ and MG (0.00518), whereas non-MG comparisons generally showed lower divergence. These elevated absolute and net divergence values indicate that the differentiation of MG was not solely attributable to reduced within-population diversity, but also reflected sequence divergence between MG and the remaining populations.

**Figure 4:**
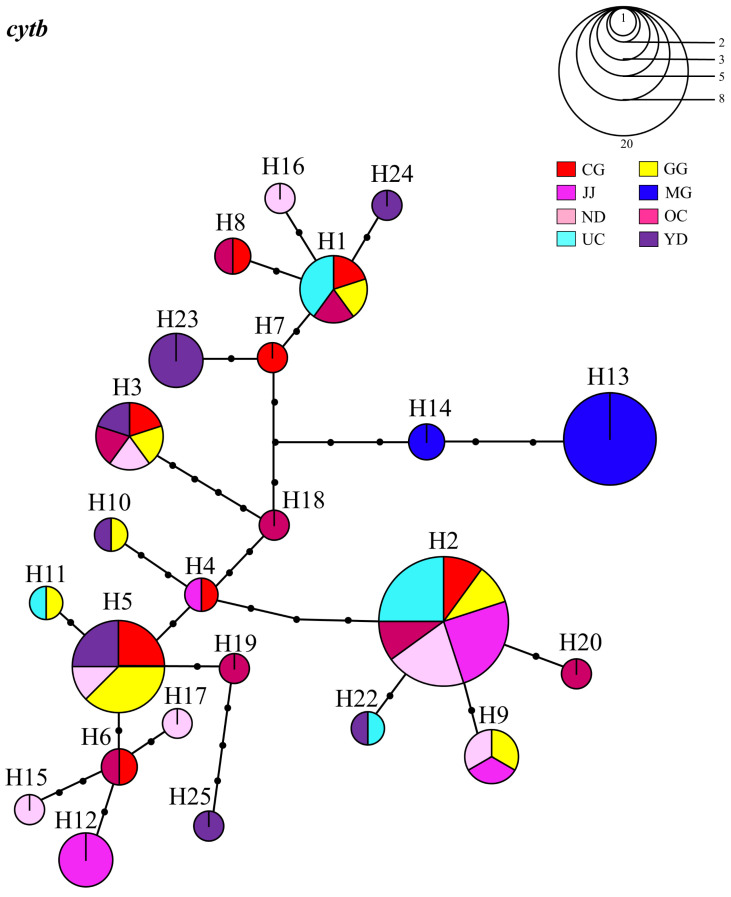
Median-joining haplotype network based on mitochondrial *cytb* sequences of *Pseudopungtungia nigra*. Each circle represents a unique haplotype, and circle size is proportional to haplotype frequency, as indicated by the scale in the upper-right corner. Colors within circles indicate the population composition of each haplotype according to the population codes shown in the legend. Small black dots on the connecting lines represent unsampled or inferred intermediate haplotypes, and each line segment between adjacent nodes represents one mutational step.

The network also showed marked differences in haplotype sharing among populations. MG contained only H13 and H14, and these haplotypes were not found in the other populations (Figure 4). By contrast, several haplotypes were shared among the non-MG populations, although their distributions differed among haplotypes. H1 was detected in CG, GG, JJ, OC, and UC; H2 in CG, GG, JJ, ND, OC, and UC; H3 in CG, GG, ND, OC, and YD; and H5 in CG, GG, ND, and YD.

**Figure 5:**
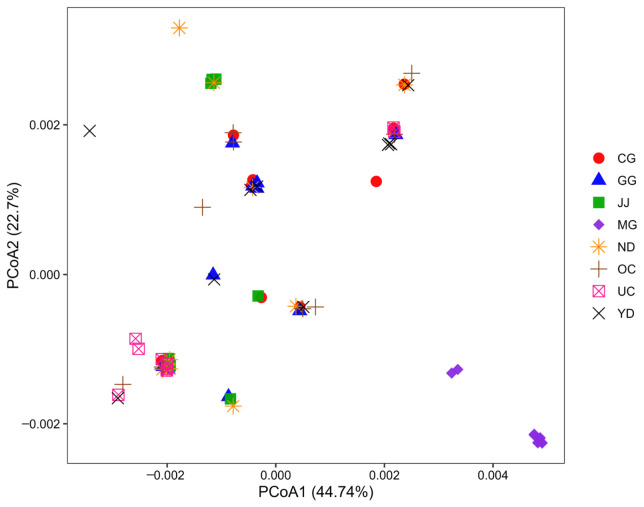
Principal coordinates analysis (PCoA) of *cytb* variation among eight populations of *P. nigra*. Principal coordinates analysis (PCoA) based on pairwise raw *P*-distances calculated from *cytb* sequences of *P. nigra*. Each point represents an individual, and colors and symbols indicate population identity. Population codes are shown in the legend at right.

The first and second axes explained 44.74% and 22.70% of the total variation, respectively (Figure 5). Individuals from MG were clearly separated from the other populations along PCoA1, whereas most non-MG populations showed partial overlap.

**Figure 6:**
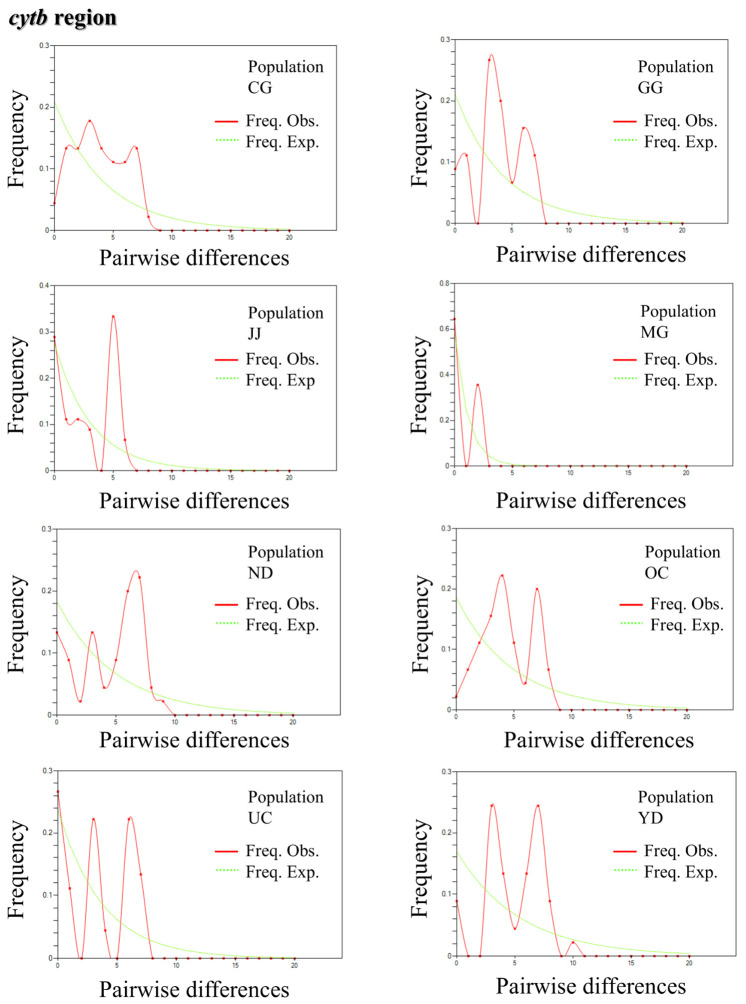
Observed and expected mismatch distributions based on *cytb* sequences for each of the eight populations of *P. nigra*. The observed distribution of pairwise nucleotide differences is shown by the red solid line, and the expected distribution under a sudden expansion model is shown by the green dotted line. A closer fit between the observed and expected curves suggests demographic expansion, whereas deviations from the expected distribution may indicate demographic stability, subdivision, or more complex population history.

Several populations, including CG, GG, ND, OC, UC, and YD, showed multimodal or irregular observed distributions that deviated from the smooth unimodal expectation under a sudden expansion model (Figure 6). JJ also exhibited a sharply uneven distribution with a pronounced peak at intermediate pairwise differences. MG showed the simplest observed pattern, reflecting its low mitochondrial variation and limited number of haplotypes.

AMOVA revealed strong hierarchical genetic structure when MG was treated as one group and the remaining populations (CG, GG, JJ, ND, OC, UC, and YD) as the other group (Table 3). Most of the total molecular variation was explained by differences among groups (56.52%), whereas only 1.68% was attributed to differences among populations within groups, and 41.80% occurred within populations. The overall fixation index was high and significant (*F*_ST_ = 0.582, *p* < 0.001), indicating substantial mitochondrial differentiation across the dataset.

## 4. Discussion

### 4.1. Genetic Diversity of P. nigra

The mtDNA cytb analysis showed that *P. nigra* retains relatively high genetic diversity at the species level, consistent with the previous microsatellite study [14]. This pattern may reflect the collective retention of different haplotypes across multiple geographically structured populations rather than uniformly high diversity within all populations [17]. In fragmented freshwater systems, restricted connectivity can allow local populations to retain different portions of historical variation, while small and isolated populations may lose haplotypes through stronger genetic drift [9,17]. In particular, several Geumgang-associated populations, including OC, CG, GG, ND, and YD, showed relatively high haplotype diversity, suggesting that cytb variation has been comparatively well preserved in these populations. This pattern is noteworthy because endangered species often show reduced genetic diversity as a consequence of small population size, drift, and habitat fragmentation [3,4]. However, relatively high species-level diversity may persist when multiple local populations collectively retain distinct lineages or when declines are too recent to have eliminated historical variation [27,36]. In this sense, the present results suggest that the endangered status of *P. nigra* does not necessarily imply uniformly low diversity across the entire species, but rather uneven retention of genetic variation among geographically structured populations.

The reduced *cytb* diversity observed in the Mangyeonggang population may reflect the effects of a small long-term effective population size, prolonged isolation, genetic drift, or past bottlenecks. By contrast, the OC population showed nine haplotypes and the highest haplotype diversity (0.978), indicating that population histories have differed substantially within the species. The reduced diversity of the MG population is also consistent with expectations for geographically isolated peripheral or drainage-restricted populations [3,17,37]. In freshwater fishes, populations confined to independent drainages often experience stronger drift and reduced opportunities for lineage exchange than populations distributed across larger or more connected systems [3,28]. Thus, the reduced *cytb* diversity of MG may be explained by alternative but non-mutually exclusive processes: a past bottleneck may have reduced haplotype richness over a relatively short period, whereas long-term isolation may have maintained a restricted haplotype composition through limited gene flow and genetic drift [17]. Because the present study used a single mitochondrial marker, these alternatives cannot be fully distinguished and should be tested further using larger sample sizes and genome-wide nuclear markers. The simplified *cytb* haplotype composition of MG may therefore reflect not only local demographic reduction, but also prolonged isolation within an independent drainage unit.

The reduced genetic diversity and strong population-specific differentiation observed in the MG population are consistent with the previous microsatellite study [14]. Specifically, MG contained only two *cytb* haplotypes, H13 and H14, which were not detected in any other sampled population, whereas several non-MG populations retained broader haplotype sharing. Thus, the reduced diversity of MG is not only a nuclear-marker pattern, but is also reflected in a restricted and population-specific mitochondrial haplotype pool.

The UC population did not show the same degree of diversity reduction as MG. This contrast suggests that geographic isolation alone does not necessarily lead to the same level of mitochondrial diversity reduction in all populations. The trend observed in UC is consistent with the previous microsatellite study, which suggested that UC has a closer genetic affinity to the Geumgang River system populations than to MG [14]. However, because the present *cytb* dataset was not designed to infer the restoration origin of UC, we interpret this pattern cautiously.

Neutrality tests were not significant in most populations, and thus the observed values of Tajima’s D and Fu’s Fs do not provide strong evidence for recent demographic expansion or contraction. However, these tests may have limited statistical power because only 10 individuals were analyzed per population and several populations had few segregating sites. Therefore, the non-significant neutrality test results should be interpreted cautiously rather than as definitive evidence of demographic stability. In particular, the reduced haplotype number and diversity in MG represent a key signal that cytb haplotypes in the Mangyeonggang population have been maintained in a comparatively restricted form.

A limitation of the present study is that only 10 individuals were analyzed per population. With such a sample size, rare haplotypes may remain undetected, and the absolute level of diversity in each population may therefore be underestimated [38,39,40]. Therefore, the observed haplotype numbers should be interpreted as minimum estimates rather than complete inventories of population-level *cytb* variation. The limitation may also bias population-level haplotype richness downward, particularly in populations where rare haplotypes occur at low frequencies. Consequently, comparisons of haplotype richness among populations may partly reflect differences in the probability of detecting rare haplotypes, although the equal sample size across populations reduces this bias in relative comparisons. In addition, neutrality tests such as Tajima’s D and Fu’s Fs may have limited statistical power under small sample sizes and low numbers of segregating sites; therefore, non-significant values should not be interpreted as strong evidence of demographic stability. Nevertheless, despite this limitation, more than four haplotypes were detected in most populations, and 25 haplotypes were identified overall. This suggests that the diversity of the species is not trivially low [25,26,41]. Moreover, because the same number of individuals was analyzed in each population, the observed differences in haplotype number and diversity are still informative in a comparative context [42,43,44]. Thus, the present dataset is best interpreted as providing evidence for relative differences among populations, even if larger sample sizes will be needed to refine estimates of absolute diversity.

### 4.2. Population Structure of P. nigra

The haplotype network identified two major mitochondrial unique haplotypes. These results indicate that the primary axis of mitochondrial structure in this species is not fine-scale subdivision among local populations, but rather the strong separation between the Mangyeonggang population and the remaining populations. The mtDNA of differentiation was stronger in the *cytb* dataset than in the previous microsatellite dataset [14]. This difference is expected because mtDNA is maternally inherited, haploid, and non-recombining, and its effective population size is approximately one-quarter that of autosomal nuclear markers under equal sex ratio assumptions [45]. Therefore, mtDNA is more sensitive to genetic drift and lineage sorting, whereas microsatellites are generally more informative for recent gene flow and contemporary nuclear population structure.

In that study, MG showed elevated pairwise *F*_ST_ values relative to the other populations, and STRUCTURE identified *K* = 2 as the optimal number of clusters, separating the Geumgang plus UC populations from the Mangyeonggang population. PCoA, DAPC, and AMOVA yielded the same general pattern [14]. The present mtDNA results therefore independently support the Geumgang vs. Mangyeonggang division reported from nuclear markers and further show that this structure is also evident at the level of lineages. This concordance suggests that the observed population structure is not specific to a single marker system, but is consistently detected across distinct genomic systems [46,47].

The inclusion of Dxy and Da further strengthens this interpretation. Absolute divergence measures are particularly informative when relative differentiation may be inflated by reduced diversity within one population, because they help distinguish sequence accumulation from diversity loss alone [48,49]. Because *F*_ST_ is a relative measure and can be influenced by reduced within-population diversity, high *F*_ST_ alone does not necessarily imply deep sequence divergence. By contrast, Dxy reflects absolute sequence divergence between populations, whereas Da reflects net divergence after correcting for within-population variation. The elevated Dxy and Da values observed in comparisons involving MG indicate that its differentiation is not simply a consequence of low internal diversity, but reflects accumulated mitochondrial sequence divergence.

The PCoA results support the same conclusion. Ordination-based approaches are useful in this context because they visualize whether the major axis of genetic differentiation is expressed consistently at the level of individual relationships, rather than only in summary statistics [50,51]. Thus, the mtDNA pattern is best interpreted as population-level differentiation of MG, rather than as complete separation among all local drainage populations.

The presence of two MG specific haplotypes indicates that the Mangyeonggang population has a restricted and population-specific *cytb* haplotype composition, rather than simply reflecting random differences in haplotype frequencies [52,53]. These results suggest long-term maintenance of a restricted lineage pool in the Mangyeonggang system.

A similar regional pattern has been reported in another Korean endemic freshwater fish, *Liobagrus geumgangensis*, which is also distributed in the Geumgang and Mangyeonggang River basins [17]. In that species, mitochondrial *cytb* data showed strong genetic differentiation among populations, including differentiation between the Geumgang and Mangyeonggang River systems [17]. Thus, the distinctiveness of the MG population in *P. nigra* is broadly consistent with a comparative phylogeographic pattern in which western Korean river systems can maintain differentiated freshwater fish populations. The differentiation of MG may reflect long-term hydrological separation of the Mangyeonggang River from the Geumgang River system, together with restricted dispersal, reduced connectivity, and stronger genetic drift [17]. However, because the present study did not examine geological history, abiotic habitat variables, or morphological, physiological, and ecological traits of each population, these possible mechanisms should be tested in future studies.

This difference is consistent with the biological properties of mtDNA, including inheritance, lack of recombination, and smaller effective population size, all of which can preserve signals of historical isolation more strongly than nuclear markers [45,54]. The Mangyeonggang population therefore appears to be differentiated in both contemporary nuclear structure and mitochondrial haplotype composition, although the present dataset is not sufficient to estimate absolute divergence time or lineage depth. In this respect, the mtDNA results add temporal depth to the population structure inferred from microsatellites. Differences in the magnitude of structure between mitochondrial and nuclear markers are not unexpected, because the two marker systems differ fundamentally in inheritance, effective population size, and sensitivity to demographic history [45,46]. Maternally inherited mtDNA can reach stronger lineage sorting under long-term isolation, whereas microsatellites are often more informative for recent connectivity and contemporary admixture. The stronger mitochondrial structure observed here is therefore consistent with a scenario in which drainage isolation has left a deeper historical signal in lineages than in biparentally inherited nuclear variation [36,52].

The position of UC is also broadly consistent between the two studies [14]. In the previous microsatellite analysis, UC showed low differentiation from Geumgang populations, and both STRUCTURE and ABC analyses supported a Geumgang origin rather than a Mangyeonggang origin. The present mtDNA results do not place UC on the same primary divergence axis as MG, which is consistent with the interpretation that the population history of UC is more closely associated with Geumgang populations than with the Mangyeonggang lineage.

As in the diversity analysis, the use of 10 individuals per population imposes some limitations on the interpretation of structure. Rare haplotypes and finer-scale within-population substructure may not be fully captured [42,43], and some relationships among populations may therefore appear more simplified than they truly are. However, despite this limitation, all overall differentiation statistics were significant, and haplotype network, PCoA, haplotype frequency patterns, and AMOVA all consistently supported the separation of MG from the remaining populations. Larger datasets will be useful for resolving finer-scale structure within the Geumgang system, but the distinctiveness of the Mangyeonggang population is already well supported by the present data.

### 4.3. Conservation Implications

The genetic distinctiveness and reduced cytb diversity of MG have important conservation implications. Because the differentiation of MG was supported by both the previous microsatellite study and the present mitochondrial cytb analysis, the Mangyeonggang population should be considered a separate conservation management unit. In particular, artificial mixing between MG and non-MG populations should be avoided unless additional genetic, ecological, and demographic evidence supports such management action.

However, this conservation interpretation does not require recognizing MG as a separate mitochondrial lineage or an evolutionarily independent taxon. Rather, the present *cytb* data indicate that MG has a restricted and population-specific haplotype composition, consistent with the population-level differentiation previously detected using nuclear microsatellite markers. Therefore, conservation strategies for *P. nigra* should preserve both species-level genetic diversity and the population-level distinctiveness of MG.

## 5. Conclusions

This study demonstrates that *Pseudopungtungia nigra* retains relatively high mitochondrial diversity at the species level, but that this diversity is unevenly distributed among populations. In particular, the Mangyeonggang population showed markedly reduced haplotype diversity and clear mitochondrial divergence from the remaining populations. This pattern was consistently supported by pairwise differentiation, Dxy and Da estimates, haplotype network analysis, PCoA, haplotype frequency patterns, and AMOVA. Importantly, the mtDNA results are concordant with previously reported microsatellite-based structure, indicating that the distinctiveness of the Mangyeonggang population is supported across both nuclear marker systems. This concordance suggests that the observed divergence reflects a historically structured population pattern rather than a marker-specific or transient signal. Taken together, these findings highlight the conservation importance of recognizing the Mangyeonggang population as a genetically distinct management unit. Future conservation and restoration strategies for *P. nigra* should therefore consider both species-wide diversity and population-level divergence in order to preserve population-level genetic distinctiveness and minimize the risk of inappropriate population mixing.

## Figures and Tables

**Table 1:** The *cytb* based genetic diversity summary information of *P. nigra*.

Group ID	*N*	*h*	*H*_d_	Nucleotide Diversity (π)	*D*	*F*	Fu’s and Li’s *D*	Fu’s and Li’s *F*
CG	10	8	0.956 ± 0.059	0.00333 ± 0.00062	−0.47046	−2.785	−0.40593	−0.47484
GG	10	7	0.911 ± 0.077	0.00329 ± 0.00063	−0.83156	−1.447	−1.14357	−1.20038
JJ	10	4	0.711 ± 0.117	0.00234 ± 0.00047	1.06453	1.258	0.77491	0.94913
MG	10	2	0.356 ± 0.159	0.00062 ± 0.00028	0.01889	1.523	1.02623	0.87315
ND	10	7	0.867 ± 0.107	0.00391 ± 0.00079	−0.72589	−1.011	−1.06037	−1.10087
OC	10	9	0.978 ± 0.054	0.00385 ± 0.00059	−0.50790	−4.031	−0.68718	−0.72376
UC	10	4	0.733 ± 0.120	0.00282 ± 0.00073	0.05623	1.731	1.02910	0.88688
YD	10	7	0.911 ± 0.077	0.00432 ± 0.00072	−0.83254	−0.769	−0.99528	−1.07596
Total of *cytb*	80	25	0.910 ± 0.021	0.00409 ± 0.00024	−0.54219	−7.413	0.89569	0.42163

*N*: number of samples, *h*: number of haplotypes, *H*_d_: haplotype diversity, *D*: Tajima’s D values, *F*: Fu’s Fs values, *D* and *F* (*p* > 0.05). Fu and Li’s *D* and *F*, neutrality test statistics based on the frequency distribution of mutations.

**Table 2:** Summary of overall genetic differentiation statistics for the *cytb* region among eight populations of *P. nigra*.

Statistic	Estimate	*p*-Value
Chi-square	260.400	0.000 ***
Hst	0.11826	0.000 ***
Kst	0.25095	0.000 ***
Kst*	0.18872	0.000 ***
Z	1182.39167	0.000 ***
Z*	6.68393	0.000 ***
Snn	0.29008	0.000 ***

Chi-square: overall chi-square test for differences in haplotype composition among populations; Hst: haplotype-frequency-based measure of population differentiation; Kst: sequence-based measure of population differentiation based on nucleotide differences; Kst*: standardized or modified sequence-based differentiation statistic; Z: Hudson’s test statistic for overall sequence differentiation among populations; Z*: modified form of Z for sequence-based population differentiation; Snn: nearest-neighbor statistic measuring the tendency of sequences to cluster with those from the same population; *p*-values were obtained from permutation tests with 50,000 replicates. ***: *p* < 0.001.

**Table 3:** Analysis of molecular variance (AMOVA) summary statistics for *P. nigra*.

Source of Variation	*d.f.*	Sum of Squares	Variance Components	Total Variance (%)	*F*-Statistics
MtDNA (CG, GG, JJ, ND, OC, UC, YD vs. MG)					
Among groups	1	43.789	2.36238	56.52	*F*_CT_ = 0.565
Among populations within groups	6	14.686	0.07004	1.68	*F*_SC_ = 0.039
Within populations	72	125.800	1.74722	41.80	*F*_ST_ = 0.582 ***
Total	79	184.275	4.17964	100.0	

*d.f*.: degrees of freedom; *** *p* < 0.001; *F*_CT_, *F*_SC_, and *F*_ST_ is based on a standard permutation across the full dataset.

## Data Availability

The *cytb* sequences were deposited in GenBank (PZ315716-PZ315740).

## Supplementary Materials

The following supporting information can be downloaded at: https://www.mdpi.com/article/10.3390/biology15110833/s1, Table S1: Sampling sites and number of individuals in the study.
