# Supplementary material for: Population Genetic Structure and Regional Divergence of the Endangered Freshwater Fish Black Shinner Pseudopungtungia nigra Based on Mitochondrial DNA
Source: Biology (Basel). 2026 May 26;15(11):833. doi: 10.3390/biology15110833 (PMC13255660; doi:10.3390/biology15110833)
Supplement: Supplementary file 1 [file biology-15-00833-s001.zip › biology-4306450-supplementary.pdf]

Table S1. Sampling sites and number of individuals in the study

Group ID	Location	Water system	*N*	Location
CG	Chogangcheon Stream	Geumgang River	10	36°13'38.69″ N, 127°48'12.56" E
GG	Geumgang Bangwuri Region	Geumgang River	10	36°01'47.35″ N, 127°38'05.98" E
JJ	Jujacheon Stream	Geumgang River	10	35°58'44.60″ N, 127°25'54.21" E
MG	Mangyeonggang River	Mangyeonggang River	10	35°58'33.61″ N, 127°12'55.02" E
ND	Namdaecheon Stream	Geumgang River	10	36°00'09.59″ N, 127°39'07.22" E
OC	Okcheon Region	Geumgang River	10	36°14'24.66″ N, 127°40'10.93" E
UC	Ungcheoncheon Stream	Mangyeonggang River	10	36°17'33.80″ N, 126°46'51.23" E
YD	Yudeungcheon Stream	Geumgang River	10	36°16'06.27″ N, 127°23'29.22" E

N: Number of samples.
